# An Effective Outcome Despite Delayed Treatment Initiation in a Female With Kallmann Syndrome: A Case Report

**DOI:** 10.7759/cureus.78864

**Published:** 2025-02-11

**Authors:** Kentaro Taniguchi, Tsukuru Amano, Akimasa Takahashi, Shunichiro Tsuji, Takashi Murakami

**Affiliations:** 1 Obstetrics and Gynecology, Omihachiman Community Medical Center, Omihachiman, JPN; 2 Obstetrics and Gynecology, Shiga University of Medical Science, Otsu, JPN

**Keywords:** bone mineral density, congenital idiopathic hypogonadotropic hypogonadism, hormone replacement therapy, kallmann syndrome, women’s health

## Abstract

Kallmann syndrome (KS) is an idiopathic hypogonadotropic hypogonadism with anosmia due to isolated gonadotropin-releasing hormone deficiency. In females, the diagnosis often occurs around age 15 due to primary amenorrhea, prompting hormone replacement therapy (HRT) initiation. However, limited data exists on the effectiveness of delayed HRT in females with KS. Here, we describe a case involving a female with KS who began treatment at the age of 27. Following HRT initiation, the patient showed increases in uterine size, bone mineral density, and growth of breasts over a two-year period. Delayed diagnosis of KS in females, as in this case, is extremely rare, and this case demonstrates two important clinical issues. First, HRT can still be effective in females with KS even if treatment initiation is delayed, and, second, therapeutic benefits become apparent within two years following initiation of treatment. These findings may be applicable to other patients with congenital hypogonadotropic hypogonadism whose treatment initiation was delayed.

## Introduction

Kallmann syndrome (KS) is an idiopathic hypogonadotropic hypogonadism with anosmia due to isolated gonadotropin-releasing hormone (GnRH) deficiency. The prevalence of KS is different between the sexes with a prevalence of approximately 1 in 30,000 males and 1 in 125,000 females [1]. It is usually diagnosed at 14-16 years of age due to delayed puberty. In males, the diagnosis may not occur until adulthood with presenting complaints of fractures or infertility [2-5]. In females, however, the diagnosis typically occurs in adolescence due to amenorrhea which prompts the initiation of hormone replacement therapy (HRT) to induce puberty as early as possible. A delayed diagnosis of KS in females is extremely rare, and there are few reports on the effectiveness of HRT in the case of delayed initiation of treatment in females. We experienced a case of a female patient with KS that remained undetected until the age of 27; however, subsequent HRT proved to be effective.

## Case presentation

A 27-year-old, gravida zero, woman was referred to our department with amenorrhea. She had never attained menarche. She had no significant past medical history, no known allergies, and no family history of similar symptoms. She had never undergone any medical assessment.

**Figure 1:**
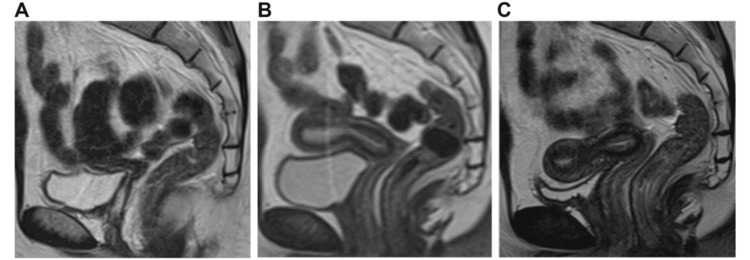
Changes in the size of the uterus. T2-weighted pelvic MRI shows uterine changes by sagittal section. The left figure represents the uterus at age 27 (initial visit), measuring 33 mm × 7 mm × 10 mm (A). The middle figure represents the uterus at age 28 (after one year of treatment), measuring 60 mm × 28 mm × 22 mm (B). The right figure represents the uterus at age 29 (after two years of treatment), measuring 70 mm × 28 mm × 23 mm (C).

**Figure 2:**
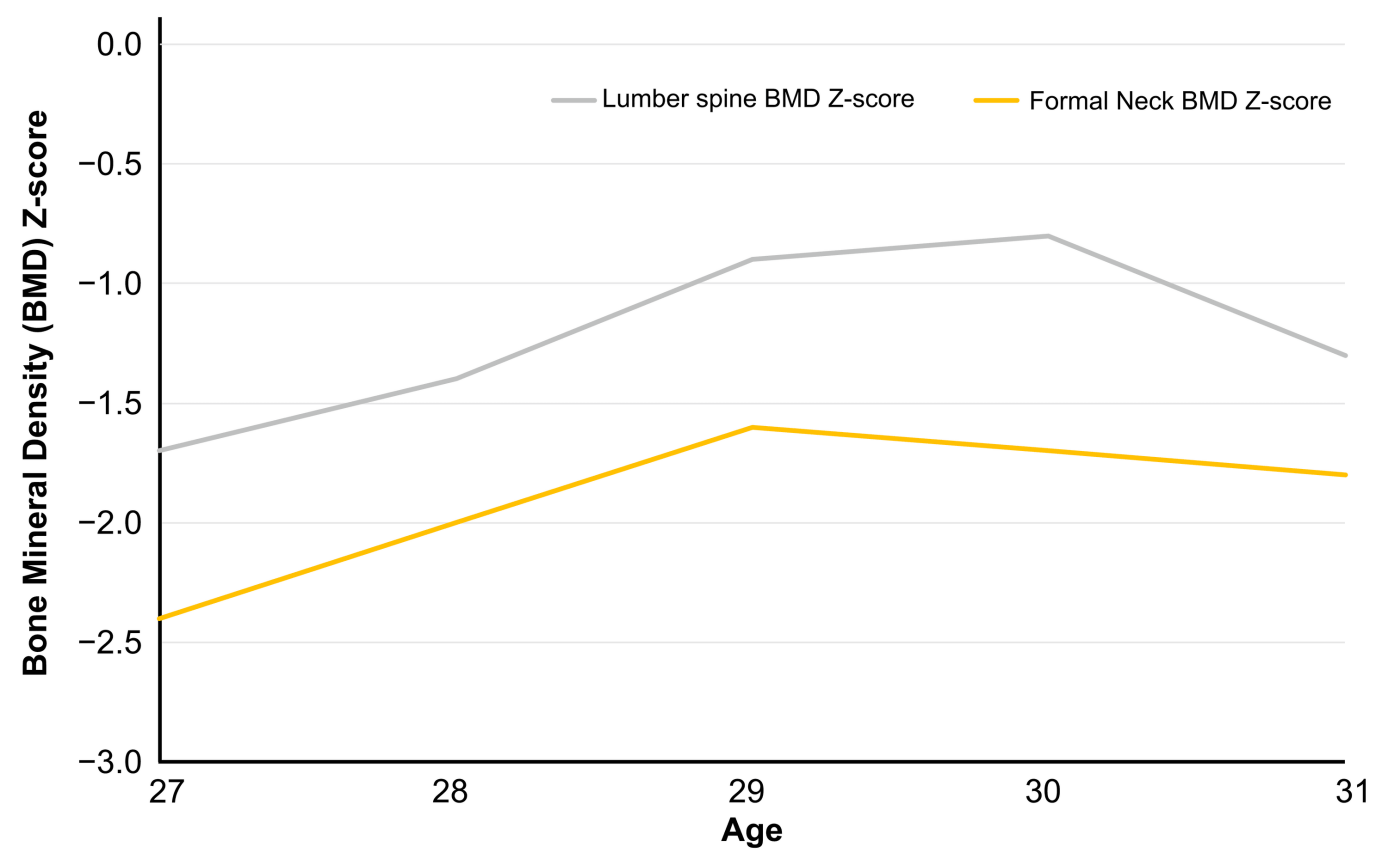
Changes in the bone density z-scores. Bone density z-scores in the lumber spine and femoral neck almost normalized after two years of treatment initiation. They gradually declined thereafter as in women without Kallmann syndrome.

Physical examination revealed the following: height, 156.0 cm; weight, 57.0 kg; body mass index, 23.4 kg/m^2^. An evaluation of her sense of smell revealed anosmia. She had a normal vagina, but a small uterus, measuring 33 mm × 7 mm × 10 mm (Figure 1A). Breast development of Stage 3 according to the Tanner stage was observed. Laboratory data found low gonadotropin levels, with follicle-stimulating hormone at 1.6 mIU/mL, luteinizing hormone at 0.4 mIU/mL, estradiol at 20 pg/mL, and progesterone at 0.2 ng/mL (Table 1). A GnRH stimulation test was performed with a positive result. MRI showed no olfactory nerve. Therefore, the diagnosis of KS was made. Her bone mineral density (BMD) was low, measuring 0.913 g/cm^2^ in the L2-L4 lumbar spine (z-score, -1.7) and 0.650 g/cm^2^ in the femoral neck (z-score, -2.4), but she had no history of fractures. We promptly initiated HRT. Estrogen was gradually increased, and breakthrough bleeding was observed to determine the appropriate dosage. The dosage was then adjusted to the following amount: day 1-10, 17β-estradiol 1 mg/day; day 11-21, 17β-estradiol 1 mg/day + dydrogesterone 10 mg/day; day 22-28, discontinuation of the medication. We continued this cycle monthly. Because there was no sexual intercourse and evaluation could not be performed using transvaginal ultrasound, changes in uterine size were tracked using MRI (Figure 1). Additionally, changes in BMD in the lumbar spine and femoral neck were monitored using dual-energy X-ray absorptiometry scans (Figure 2). After one year of treatment, the uterus measured 60 mm × 28 mm × 22 mm (Figure 1B), while the BMD in the L2-L4 lumbar spine measured 0.948 g/cm^2^ (z-score, -1.4), and in the femoral neck 0.694 g/cm^2^ (z-score, -2.0). After two years of treatment, the uterus measured 70 mm × 28 mm × 23 mm (Figure 1C), while BMD in the L2-L4 lumbar spine measured 1.013 g/cm^2^ (z-score, -0.9) and in the femoral neck measured 0.740 g/cm^2^ (z-score, -1.6). Breast development of Stage 3 according to the Tanner stage was observed. As the uterine size had become normal, we focused primarily on monitoring BMD. After three years of treatment, BMD in the L2-L4 lumbar spine measured 1.025 g/cm^2^ (z-score, -0.8), and in the femoral neck 0.734 g/cm^2^ (z-score, -1.7). After four years of treatment, BMD in the L2-L4 lumbar spine measured 0.969 g/cm^2^ (z-score, -1.3), and in the femoral neck 0.721 g/cm^2^ (z-score, -1.8).

**Table 1:** Pituitary and gonadal hormones at presentation. This reference range is for premenopausal females. LH: luteinizing hormone; FSH: follicle-stimulating hormone

	Results	Reference range
LH (mIU/mL)	0.4	1.8–88.33
FSH (mIU/mL)	1.6	1.47–16.6
Prolactin (ng/mL)	7.19	4.91–29.3
Estradiol (pg/mL)	>5.0	28.8–525.9
Progesterone (ng/mL)	0.2	0–24.2
Testosterone (ng/mL)	17.2	0.11–0.47

## Discussion

In this patient, the delayed diagnosis of KS was attributed to a lack of recognition of symptoms such as anosmia and amenorrhea. We were able to identify two crucial clinical findings. First, in females with KS, HRT is effective even with delayed treatment initiation, and, second, uterine size and BMD in the lumbar spine and femoral neck reach approximately normal levels after two years of treatment.

The first finding underscores the continued efficacy of HRT in females with KS who experience delayed treatment initiation. Early diagnosis and induction of puberty in congenital hypogonadotropic hypogonadism (CHH), including KS, has been shown to be advantageous for sexual, skeletal, and metabolic health, and potentially mitigates the psychological impact of CHH [6-8]. While the ideal age for initiating treatment in CHH remains undetermined, prompt initiation is advised [7]. Although KS is occasionally diagnosed in males during adulthood due to fractures, in females, it is commonly identified around age 15 due to the absence of menarche, prompting immediate therapeutic intervention. Only a few cases have been described where females commenced treatment in adulthood leaving the efficacy of such treatment unclear [9]. However, this case illustrates the potential effectiveness of treatment initiated during adulthood in women with KS.

The second finding underscores that after a two-year treatment period, both uterine size and BMD in the lumbar spine and femoral neck were approximately within the normal range. The impact of HRT on KS patients has not been definitively established due to few case studies. There is a significant correlation between 17β-estradiol dosage and uterine size for pubertal induction, but the optimal, effective HRT regimen to maximize reproductive potential without adverse effects remains unclear [10]. In Turner syndrome (TS), high-dose oral 17β-estradiol (4 mg/day) induces a more rapid increase in uterine size within the first years of treatment compared with a low dose (2 mg/day). However, the uterine growth potential appears to be the same in youngest women with TS making the duration of treatment as important as the estrogen dose [11]. If this assessment is applicable to the patient in this case, a higher 17β-estradiol dosage could have induced a steeper increase in uterine size. However, given the fact that she eventually obtained a healthy uterine size, the dosage and duration of estrogen administration were appropriate. In terms of BMD, males undergoing adequate HRT for over two years exhibited a higher BMD compared to those treated for fewer than six months [12]. This may also apply to women with KS.

## Conclusions

This case demonstrates that HRT is effective even in delayed treatment initiation for KS. Properly monitored therapy can lead to normal uterine development and improved bone health, emphasizing the importance of recognizing KS symptoms early to initiate timely intervention. Therapeutic benefits become apparent within the initial two-year period and persist consistently thereafter. These findings may be applicable to other cases of CHH with delayed treatment initiation.
